# Breeding potential of *Aedes albopictus* (Skuse, 1895) in chikungunya affected areas of Kerala, India

**Published:** 2010-12

**Authors:** Alex Eapen, K. John Ravindran, A.P. Dash

**Affiliations:** * National Institute of Malaria Research (ICMR) Field Unit, NIE campus, 2^nd^ Main Road, TNHB, Ayapakkam, Chennai 600 077, India; ** World Health Organization, Regional Office for South East Asia, Indraprastha Estate Mahatma Gandhi Marg, New Delhi 110 002, India

Sir,

In India, chikungunya re-emerged after a lapse of three decades in a virulent epidemic form in late 2005. In 2006, a total of 1.39 million suspected cases from 213 districts in 15 States and about 565.42 million people were at the risk of infection^1^–^4^. There were 70,731 suspected cases in Kerala state during 2006 mainly from 3 coastal districts *viz*., Alappuzha with a maximum of 58,308 (82.44%), Thiruvananthapuram with 8,311 (11.75%) and Eranakulam with 1,840 (2.60%) cases mostly confined to urban areas including small townships^3^. In 2007, Kerala was the worst affected State in India with a recorded incidence of 24052 suspected cases of which 909 were confirmed cases^5^, almost exclusively rural mainly in 6 districts in the central and southern parts of Kerala. Among these districts, Kottayam and Pathanamthitta were the worst affected, contributing 44.33 and 14.37 per cent of the total cases respectively^3^. We studied prevalence of different species of *Aedes* in chikungunya-affected areas and their breeding potential in June 2007 after the outbreak.

The criteria for selection of the districts was based on the high incidence of confirmed cases recorded by the health department of Kerala. Every 4^th^ house in the affected ward was visited to ascertain the breeding potential. Immature population density was recorded using a standard dipper (300 ml with 9 cm diameter) in larger containers. In smaller containers, the sampling was performed using a dipper of 150 ml capacity with 6.5 cm diameter. Container breeding in peridomestic and intradomestic areas were qualitatively and quantitatively assessed. The ornamental plants of peri-domestic areas and plantations of pineapple were surveyed to estimate the extent of plants associated with breeding of Aedes in the chikungunya affected wards. Besides, breeding of Aedes in rubber plantations characteristic of some of the districts was also surveyed. The immature of both larvae and pupae collected were scored and subsequently allowed to hatch for identification of the mosquito species. Entomological parameters such as the house index (HI), container index (CI), breteau index (BI) and pupal index (PI) were estimated by the standard procedures^6^. Adult landing collection of mosquitoes were also undertaken and scored for Aedes mosquitoes.

In Thiruvanathapuram, Pathanamthitta and Kottayam districts, Aedes breeding was observed in discarded coconut shells and plastic containers in rubber plantations. The metallic/plastic containers located at the base of the refrigerator in a few houses (Pathanamthitta and Kottayam districts) also supported breeding of *Ae. albopictus*. The adults emerged from the immatures collected during the survey indicated predominance of *Ae. albopictus* with a total number of 287 (75.3%) followed by *Ae. vittatus* (24.1%). Adult collections also revealed the exclusive presence of *Ae. albopictus* with density ranging from 10 to 26 per hour. All the 10 PHCs surveyed had high BI and PI except Pazhavangadi PHC in Pathanamthitta district (Table). Analysis of the HI in the surveyed areas indicated an overall significant difference (Chi square, *P*=0.01). However, there is no significant difference in breeding observed in houses between any of the two districts (multiple comparison by Bonferroni correction factor). Similarly, the CI also revealed an overall significant difference in breeding observed in the surveyed areas (*P*<0.001).

**Table:** Immature survey in the affected PHCs of Kerala

Name of PHC (District)	Ward Nos.	HI	CI	BI	PI
Vellarada (Thiruvananthapuram)	2	49.2	49.7	157.4	157.1
	5	30.7	20.5	44.4	80.8
Perinad	1	30.8	30.5	96.2	150
	14	36.4	9.3	36.4	36.4
Seethathodu Pazhavangadi (Pathanamthitta)	6	27.3	17.6	54.5	9.1
	11	9.5	8.7	9.5	0
Thidanadu	7	22.2	9.8	55.6	133.8
	8	36.8	12.4	73.7	10.5
T.V. puram (Kottayam)	11	46.7	29.5	110	23.3
Kuttanpuzha	8	31.3	10.6	43.8	0
	9	22.2	8.9	27.8	5.5
	12	50	20	72.7	0
Maradi (Eranakulam)	10	61.1	34	94.4	186.1
Methala (Thrissur)	12	20.8	2.8	32.5	41.2
	13	14.8	1.6	18.5	63
Puthusserry (Palakkad)	6	32.3	24.6	47.5	53.2
	7	28.6	13.8	52.4	52.4

HI, house index; CI, container index; BI, breteau index; PI, pupal index

Ae. albopictus breeding was encountered in the leaf axils of many plants *viz*., pineapple (*Ananas comosus*), bromeliads (*Guzmania* spp), banana (*Musa* spp), screw pine (*Pandanus odoratissimus*) and in the primary rachis of coconut (*Cocos nucifera*). Breeding of *Ae. albopictus* in plants observed in the affected areas ranged from 7.7 to 70.4 per cent. Maximum *Ae. albopictus* breeding in plants were recorded in Thiruvananthapuram followed by Pathanamthitta, Kottayam, Palakkad, Eranakulam, and Palakkad districts. Among the different plants, 80.8 per cent of breeding was observed in the leaf axils of pineapple, followed by flowering plants (7.8%), 5 per cent each in screw pine and primary rachis of coconut palm trees and 1.45 per cent in banana plants.

The attack rates observed in the survey areas ranged from 23.18 (Thiruvananthapuram) to 57.5 (Kottayam). Absence of herd immunity in the affected population can be considered as one of the reasons for such a large outbreak^7^. The present field study clearly indicated that peridomestic container breeding as well as rubber plantations largely supported the population build up of *Ae. albopictus* in these areas as revealed by the high PI observed. Sylvan environment of rubber plantations has been detected as a unique habitat of *Ae. albopictus* as also seen in the present study^8^–^10^.

*Ae. aegypti* is regarded as the primary vector for chikungunya. Nevertheless, the outbreak of chikungunya in Reunion Island and many other island countries during 2005-2006 indicated mutation in chikungunya virus, which facilitated its transmission by *Ae. albopictus*^11^,^12^. Similarly the outbreak of chikungunya in Italy is attributed to *Ae. albopictus*^13^. The enhanced chikungunya virus infection of *Ae. albopictus* was reported to be due to point mutation in one of the viral envelope genes - E1^14^. The extensive present survey could not detect *Ae. aegypti*, which suggests the possibility of the role of *Ae. albopictus* as the vector of chikungunya. The epidemic, which occurred during 2005-2006 in certain islands of Indian Ocean and in Kerala, indicated the probable role played by *Ae. albopictus*^15^,^16^. Isolation of chikungunya virus from *Ae. albopictus* in Kerala is yet to be demonstrated. Similar survey carried out in Lakshadweep islands, Indian Ocean during November/December 2006, which experienced chikungunya, revealed the predominance of *Ae. albopictus* and absence of *Ae. aegypti*^17^.

A significant finding in the present investigation is the role of certain species of plants serving as an ideal habitat conducive for breeding of *Ae. albopictus* as has been reported from Thailand^18^. The present data indicated that pineapple plants support maximum breeding of *Ae. albopictus*, and Kerala has extensive pineapple plantations. The versatile adaptability of *Ae. albopictus* to switch over to breeding in plants poses a serious problem to control vector breeding, as source reduction of vector is the recognised strategy for the control of chikungunya fever. The present study indicated the preponderance of *Ae. albopictus* in the affected wards/districts of Kerala with chikungunya, resorting to breeding in containers and certain species of plants. Phylogenetic analysis indicated that the virus strain that caused outbreak in Indian subcontinent contained the same mutation, which is thought to be better adapted to *Ae. albopictus*^13^. Novel strategies to eliminate prolific breeding of *Ae. albopictus* need to be developed besides, strengthening source reduction methods involving community to control future chikungunya outbreaks.

## References

[1] (2007). National Vector Borne Disease Control Programme. Chikungunya fever situation in the country during 2006 (Prov.), NVBDCP, Government of India.

[2] Kumar NP, Madhu Mitha M, Krishnamoorthy N, Kamaraj T, Joseph R, Jambulingam P (2007). Genotyping of virus involved in the 2006 Chikungunya outbreak in South India (Kerala and Puducherry). *Curr Sci*.

[3] Kumar NP, Joseph R, Kamaraj T, Jambulingam P (2008). A226V mutation in virus during the 2007 chikungunya outbreak in Kerala, India. *J Gen Virol*.

[4] Krishnamoorthy K, Harichandrakumar KT, Krishna Kumari A, Das LK (2009). Burden of chikungunya in India: estimates of disability adjusted life years (DALY) lost in 2006 epidemic. *J Vector Borne Dis*.

[5] (2008). National Vector Borne Disease Control Programme. Dengue/ DHF. NVBDCP, Government of India.

[6] (1999). World Health Organization. *Prevention and control of dengue and dengue haemorrhagic fever,* 29.

[7] Mourya DT, Mishra AC (2006). Chikungunya fever. *Lancet*.

[8] Hiriyan J, Tewari SC, Tyagi BK (2003). *Aedes albopictus* (Skuse) breeding in plastic cups around tea vendor spots in Ernakulam city, Kerala state, India. *Dengue Bull*.

[9] Sumodan PK (2003). Potential of rubber plantations as breeding source for *Aedes albopictus* in Kerala, India. *Dengue Bull*.

[10] Kalra NL, Prasittisuk C (2004). Sporadic prevalence of DF/DHF in the Nilgiri and cardamom hills of Western ghats in South India: Is it a seeding from sylvatic Dengue cycle - A hypothesis. *Dengue Bull*.

[11] Tsetsarkin KA, Vanlandingham DL, McGee CE, Higgs S (2007). A single mutation in chikungunya virus affects vector specificity and epidemic potential. *PLosPathog*.

[12] Townson H, Nathan MB (2008). Resurgence of chikungunya. *Trans R Soc Trop Med Hyg*.

[13] Rezza G, Nicoletti L, Angelini R, Romi R, Finarelli AC, Panning M (2007). Infection with chikungunya virus in Italy: an outbreak in a temperate region. *Lancet*.

[14] Enserink M (2007). Epidemiology. Tropical disease follows mosquitoes to Europe. *Science*.

[15] chuffenecker I, Iteman I, Michault A, Murri S, Frangeul L, Vaney MC (2006). Genome microevolution of Chikungunya viruses causing the Indian Ocean outbreak. *PloS Med*.

[16] Santhosh SR, Dash PK, Parida MM, Khan M, Tiwari M, Lakshmana Rao PV (2008). Comparative full genome analysis revealed E1: A226V shift in 2007 Indian Chikungunya virus isolates. *Virus Res*.

[17] Samuel PP, Krishnamoorthi R, Hamzakoya KK, Aggarwal CS (2009). Entomo- epidemiological investigations on chikungunya outbreak in the Lakshadweep islands, Indian Ocean. *Indian J Med Res*.

[18] Preechaporn W, Jaroensutasinee M, Jaroensutasinee K (2006). The larval ecology of Aedes aegypti and *Ae. albopictus* in three topographical areas of Southern Thailand. *Dengue Bull*.

